# A novel insight into ComE-mediated activation of gene expression in *Streptococcus mutans*

**DOI:** 10.1128/spectrum.01477-25

**Published:** 2025-07-07

**Authors:** Hemendra Pal Singh Dhaked, Saswati Biswas, Indranil Biswas

**Affiliations:** 1 Department of Microbiology, Molecular Genetics, and Immunology, University of Kansas Medical Center 21638, Kansas City, Kansas, USA; University of Manitoba, Winnipeg, Manitoba, Canada

**Keywords:** *Streptococcus mutans*, two-component regulatory systems, oral streptococci, ComD/ComE, bacteriocins

## Abstract

Bacteria use two-component signal transduction systems (TCSs) to sense and respond to environmental changes. TCSs comprise a histidine kinase that senses environmental signals and a response regulator that regulates cognate promoters. Most response regulators are incapable of regulating their promoters in the absence of the cognate histidine kinases. Here, we demonstrated that constitutive expression of the well-studied response regulator ComE of *Streptococcus mutans* (a dental pathogen) activates the *nlmA* promoter (P*nlmA*) for bacteriocin production without the involvement of its cognate histidine kinase ComD. To confirm the phosphorylation-independent role of ComE, we generated a phospho-mimicking (D60E) and a phospho-inactivating (D60A) variant and examined their ability to activate P*nlmA* in a Δ*comD* knockout strain. We found that the phospho-mimicking D60E variant was able to induce the P*nlmA* expression. However, we also found that the phospho-inactivating variant also activates P*nlmA*. Furthermore, an *in vitro* binding assay suggested that both the phospho-mimicking and the phospho-inactivating variants were able to bind to P*nlmA*. Using a Phos-Tag gel analysis, we confirmed that the D60 of ComE is responsible for phosphorylation. Overall, this study demonstrated the autonomous role of the response regulator ComE by modulating the expression of its cognate promoters. We believe that other streptococcal ComE homologs might activate their target genes in a phosphorylation-independent manner similar to *S. mutans* ComE.

**IMPORTANCE:**

Bacteria utilize two-component signal transduction systems to detect and respond to environmental changes, typically involving a histidine kinase and a response regulator. While most response regulators need their cognate histidine kinases to modulate gene expression, our study shows that the response regulator ComE in *Streptococcus mutans,* a dental pathogen*,* can activate bacteriocin production without its cognate histidine kinase ComD. This study indicates that ComE can autonomously modulate promoter expression, suggesting similar mechanisms in other streptococcal ComE homologs.

## INTRODUCTION

*Streptococcus mutans* is a primary causative pathogen of dental plaque (tooth decay) on human teeth (1–3). The dental plaque biofilm contains ~700 bacterial species (4–7). Dental biofilms are exposed to various adverse environmental factors, such as pH change, lack of nutrient availability, and change in redox potential. The constant changes in the environment influence the microbial population in dental biofilm (2, 3, 8). However, *S. mutans* adapt, tolerate, and respond to these adverse environmental conditions and become the predominant species of the biofilm community. Two-component signal transduction systems (TCSs) are one of the major mechanisms by which bacteria sense and respond to such adverse conditions. The majority of the TCS consists of a membrane-bound histidine kinase (HK) and a response regulator (RR). The membrane-bound N-domain of HK senses signals from the extracellular environment, while the cytoplasmic C-domain relays the signal to the RR for the modulation of gene expression. In general, the signals are unknown; however, in some cases, quorum-sensing signals are auto-inducing peptides. These peptides are ribosomally synthesized and processed through dedicated exporters/transporters. These exporters cleave peptides at the conserved leader sequence and release their mature active form in the extracellular environment. As the bacterial population density increases, the secreted mature peptide accumulates, and when it reaches above a threshold concentration, the peptide activates the cognate HK. The HK then phosphorylates itself by receiving a phosphoryl group from ATP. Subsequently, HK accelerates the intrinsic ability of the cognate RR, leading to its phosphorylation. The RR in bacteria generally comprises two domains: a phospho-receiver domain with a conserved aspartate residue and an effector domain that binds to DNA upon phosphorylation of the aspartate residue. A phosphorylated RR interacts with cognate promoters and activates or represses their expression (9–13). In the majority of the cases, RRs depend on their cognate HK for phosphorylation and signal transduction (14). In some cases, such as the PhoP/PhoQ TCS, the PhoP RR can activate target genes without the PhoQ HK (15). In other cases, some RRs are orphan, such as CovR, which modulates its target promoters in both phosphorylated and non-phosphorylated states (16). Sometimes a single mutation in RRs can also affect their ability to activate the cognate promoter. Nicod et al. (17) investigated the amino acid residues of the LytTR domain of AgrA present near DNA and found that mutants L171A, E181A, and H200A impair the DNA-binding activity of AgrA, resulting in transcription activation of the *agr* operon.

*S. mutans* encodes 14 different TCSs, which are required for adaptation, bacteriocin production, and biofilm formation (18–20). Among them, the ComDE (also known as BlpRH) is a highly conserved TCS across the genus *Streptococcus*. In *S. mutans,* the ComDE system regulates genetic competence and bacteriocin production (21). The primary function of bacteriocin secretion is to inhibit the growth of other competing streptococcal species in the colony niches (22, 23). The ComDE homologs in *Staphylococcus aureus* (AgrAC) and *Streptococcus pneumoniae* (BlpRH) are very well studied in terms of biological function and biochemical and structural characterizations (24–30). In contrast, very few biochemical and functional studies have focused on the ComDE system from *S. mutans* (31, 32). In our previous study, we observed that purified unphosphorylated ComE (RR) can interact with the cognate *nlmA* promoter (P*nlmA*) (33). This observation prompted us to further investigate the role of phosphorylation of ComE for its biological activity. In this study, using a P*nlmA-gusA* reporter construct, we examined the ability of ComE to activate the P*nlmA* promoter. We constructed the wild-type (WT) ComE, a phospho-mimicking variant of ComE (D60E), and a non-phosphorylating variant of ComE (D60A) and tested their ability to activate the P*nlmA* promoter in isogenic Δ*comD* and Δ*comE* background strains. Surprisingly, we observed that the wild-type ComE activated the P*nlmA* promoter even in the Δ*comD* strain. We also observed that the phospho-mimicking variant enhanced the P*nlmA* expression in both the Δ*comD* and Δ*comE* strains. Surprisingly, we observed that the non-phosphorylating variant, ComE D60A, also activated P*nlmA* expression in both Δ*comD* and Δ*comE* reporter strains. These results indicated that ComE can activate its cognate promoter *in vivo* without phosphorylation. This is the first report exploring the phosphorylation-independent role of ComE and its homologs in the activation of target genes.

## MATERIALS AND METHODS

### Bacterial strains, plasmids, and growth conditions

Bacterial strains and plasmids used in this study are listed in Table 1. *Escherichia coli* DH5α or *E. coli* BL21(DE3) cells were grown in Lysogeny Broth (LB) medium with appropriate antibiotics (100 µg/mL ampicillin [Ap] or 500 µg/mL erythromycin [Em]). *E. coli* cells were grown at 37*°*C under shaking conditions. Streptococcal strains were grown in Todd-Hewitt medium supplemented with 0.2% yeast extract (THY medium) containing 300 µg/mL kanamycin (Km) and 10 µg/mL Em, whenever required. For transformation, *S. mutans* cells were grown until the OD_600_ reached 0.2. Then, competence-stimulating peptide (CSP) 18 peptide (400 nM) was added to the culture, followed by a 10 min incubation. CSP18 was bought from GenScript and had 95% purity. Afterward, 500 ng of DNA was added to the culture and incubated for 2 h. Cultures were plated on THY-agar plates with appropriate antibiotics and incubated at 37*°*C in a candle jar.

**TABLE 1:** List of strains and plasmids used in this study

Strain or plasmid	Description	Source
Strains		
UA159	Wild type	(34)
IBS1C14	UA159::P*nlmA-gusA* reporter, Km^r^	(33)
IBSN39	UA159::Δ*comE* (markerless deletion of *comE*)	(33)
IBS1C15	Δ*comE*::P*nlmA-gusA* reporter, Km^r^	(33)
IBS1C61	Δ*comD*::P*nlmA-gusA* reporter, Km^r^	This study
*E. coli* DH5α	Cloning strain	
Lab stock *E. coli* BL21(DE3)	Protein expression with T7 promoter	
Lab stock		
Plasmids		
pIB184Em	*E. coli*-streptococcal shuttle plasmid, P23 promoter, Em^r^	(35)
pET15b	Expression plasmid, Ap^r^	Novagen
pIB1B2	pET15b with His-ComE, Ap^r^	(33)
pIB1B87	pET15b with His-ComE D60A, Ap^r^	This study
pIB1B88	pET15b with His-ComE D60N, Ap^r^	This study
pIB1B89	pET15b with His-ComE D60E, Ap^r^	This study
pIB1E6	pET15b with His-ComE H201A, Ap^r^	This study
pIB1B68	pIB184Em with ComE, Em^r^	(33)
pIB1B37	pIB184Em with *S. pneumoniae* BlpR, Em^r^	(33)
pIB1B99	pIB184Em with *S. aureus* AgrA, Em^r^	(33)
pIB1E1	pIB184Em with *Streptococcus gallolyticus* BlpR, Em^r^	(33)
pIB1E2	pIB184Em with *Streptococcus sobrinus* BlpR, Em^r^	(33)
pIB1B96	pIB184Em with ComE D60A, Em^r^	This study
pIB1B97	pIB184Em with ComE D60N, Em^r^	This study
pIB1B98	pIB184Em with ComE D60E, Em^r^	This study
pIB1E11	pIB184Em with ComE D60A/E62A/E66A/E67A/E72A, Em^r^	This study
pIB1E12	pIB184Em with ComE D60A/E66A, Em^r^	This study
pIB1E13	pIB184Em with ComE H201A, Em^r^	This study

### Construction of ComE variants for purification and complementation

Purification of ComE was performed using *E. coli* BL21 (DE3) cells containing pIB1B2 (pET15b with ComE) as described in our previous study (33). To prepare ComE mutants D60E, D60A, and H201A, site-directed mutagenesis was performed. Complementary forward and reverse primers were designed as listed in Table 2. PCR amplification of these mutants was performed using pIB1B2 as a template. The amplified PCR products were digested with *Dpn*I and then transformed into *E. coli* DH5a cells. The tentative plasmid clones were isolated and confirmed by DNA sequencing. These plasmids were named as pIB1B87 (D60A), pIB1B89 (D60E), and pIB1E6 (H201A).

**TABLE 2:** List of primers used in this study

Primer name	Sequence (5′- 3′)
BamHI-rbsComE-F	CGCGGATCCAAGAAGGAGGATATACAAATGATTTCTATTTTTGTATTGG
XhoI-ComE-R	CCGCTCGAGTCATTTTGCTCTCCTTTGATCAGCAATC
ComE-D60A-F	CCAGATTTTCTTTTTGGCTATTGAAATCAAAAAAGAGG
ComE-D60A-R	CCTCTTTTTTGATTTCAATAGCCAAAAAGAAAATCTGG
ComE- D60N-F	CCAGATTTTCTTTTTGAATATTGAAATCAAAAAAGAGG
ComE- D60N-R	CCTCTTTTTTGATTTCAATATTCAAAAAGAAAATCTGG
ComE- D60E-F	CCAGATTTTCTTTTTGGAAATTGAAATCAAAAAAGAGG
ComE- D60E-R	CCTCTTTTTTGATTTCAATTTCCAAAAAGAAAATCTGG
ComEE62A/E66A/E67A/E72A-F	GGCTATTGCAATCAAAAAAGCGGCAAAGAAAGGACTGGCAGTAGCC
ComE-E62A/E66A/E67A/E72A-R	GGCTACTGCCAGTCCTTTCTTTGCCGCTTTTTTGATTGCAATAGCC
ComE-E66A-F	GAAATCAAAAAAGCGGAAAAGAAAGG
ComE-E66A-R	CCTTTCTTTTCCGCTTTTTTGATTTC
ComE-H201A-F	GATAAGAGACTTTTTCAGTGCGCACGCTCTTTTATTGTCAATCC
ComE-H201A-R	GGATTGACAATAAAAGAGCGTGCGCACTGAAAAAGTCTCTTATC
Biotin-pnlmA-F	TACAAATATGGCAATCGAAG
pnlmA-R	TCAAATGCCTGTGTATCCAT

For complementation studies, previously designed pIB1B68 (pIB184Em with wild-type *S. mutans comE*) was used (33). The variants of *comE* were cloned into pIB184Em using primers BamHI-rbscomE-F and XhoI-comE-R (Table 2) with appropriate pET15b derivatives as template. The resultant plasmids were confirmed by DNA sequencing and named as pIB1B96 (D60A), pIB1B98 (D60E), and pIB1E13 (H201A). Similarly, other variants D60N, D60A/E62A/E66A/E67A/E72A, D60A/E66A were prepared using appropriate templates and named pIB1B97 (D60N), pIB1E11 (D60A/E62A/E66A/E67A/E72A), and pIB1E12 (D60A/E66A), respectively. These plasmids were transformed into *S. mutans* Δ*comE* or Δ*comD* strains separately.

### β-glucuronidase assay

β-glucuronidase (Gus) assay was performed to measure the effects of ComE and its variants on P*nlmA-gusA* in *S. mutans* UA159, Δ*comE,* and Δ*comD* reporter strains (33). Briefly, overnight-grown cells were diluted (1:20) in THY medium and grown until the OD_600_ reached ~0.6 at 37*°*C under static conditions. Three milliliters of culture was harvested and stored at −20*°*C. The pellet was resuspended in 600 µL of Z-buffer (60 mM Na_2_HPO_4_, 40 mM NaH_2_PO_4_, 10 mM KCl, and 1 mM MgSO_4_). In the resuspended culture, 40 µL of freshly prepared 10 mg/mL lysozyme, 8 µL of 10% (vol/vol) Triton X-100, and 20 mM DTT were added and incubated at 37°C for 30 min. Then, 200 µL of *p*-nitrophenyl-D-glucoside (at 4 mg/mL in Z buffer) was added and again incubated at 37°C for 40 min. The reaction was stopped by the addition of 400 µL of 1 M Na_2_CO_3_. The reaction mixture was centrifuged, and the OD_420_ of the supernatant was measured. The Gus activity was calculated as (1,000 × OD_420_)/(time in minutes × OD_600_) in Miller units.

### Purification of ComE and its variants

Purification of ComE and its variants was performed using a similar method as described in our previous study. Briefly, pET15b derivatives containing *comE* and its variants with histidine tag were transformed into *E. coli* Bl21 cells. The cells were grown in 2× yeast extract with tryptone at 37°C with shaking (220 rpm) until the OD_600_ reached ≈0.6. The cells were kept at 18°C for 30 min with slow shaking (150 rpm) and induced with 0.5 mM IPTG for 18 h. The cells were harvested, lysed, and processed for purification as described previously (33). Protein samples were stored at –80°C in 20 mM Tris, pH 7.4, 200 mM KCl, and 30% (vol/vol) glycerol. Proteins were briefly spun at 18,407 rcf for 10 min at 4°C before each experiment. The concentration of protein was measured by Bradford’s reagent using BSA as a standard (36).

### Biolayer interferometry for DNA-protein interaction

Biolayer interferometry (BLI) was performed to observe the interaction of ComE and its variants with the *nlmA* promoter, as described previously (33). Briefly, P*nlmA* (~500 bp) was tagged at the 5′ end using a biotinylated primer (Table 2) during PCR amplification. The amplified PCR product was purified and diluted to 40 ng/µL in binding buffer [20 mM Tris, 50 mM KCl, 0.01 mM DTT, 5% glycerol (vol/vol), 1 mM EDTA, 0.01 mg/mL BSA, 5 mM MgCl_2_, and 10 µg/mL poly(dI-dC) at pH 7.4]. The biotin-P*nlmA* was loaded on a hydrated streptavidin biosensor tip for 5 min. The biosensor tip was washed with a binding buffer for 2 min. The association reaction of biotin-P*nlmA* with 1 µM protein (incubated with binding buffer) was performed for 5 min. Experiments were repeated three times.

### Phos-tag SDS-PAGE analysis

Phos-tag SDS-PAGE was performed to observe differences between phosphorylated and non-phosphorylated forms of proteins as described previously (16). Eight micrograms of purified ComE (WT) and its variants (D60E and D60A) were incubated in buffer containing 20 mM Tris (pH 7.4), 20 mM MgCl_2_, 50 mM KCl, 5 mM DTT, and 10% (vol/vol) glycerol for 1 h on ice. Then, 200 mM PAM (synthesized at KU; >90% purity) was added and again incubated for 1 h on ice to maintain the stability of ComE and the phosphoryl group. When needed, samples were heated at 95°C for 5 min before loading onto a 12% Phos-tag SDS-PAGE gel (29:1 acryl:bis-acryl, 75 µM phos-tag, and 200 µM MnCl_2_) and electrophoresed at 4°C for 5 h at 90 V. Experiments were repeated at least twice.

### Absorbance kinetics

The absorbance kinetics was determined to monitor the polymerization effects of magnesium chloride on ComE and its variants. Proteins at a concentration of 4 µM were incubated in a buffer containing 20 mM Tris (pH 7.4), 50 mM KCl, and 5 mM DTT for 1 h on ice. Subsequently, reaction mixtures were incubated without or with 10 mM MgCl_2_, mixed thoroughly, and immediately transferred to a multiplate reader (Tecan Spectramax). The turbidity was measured for 20 min at *A*_350 nm_ (37–39). The absorbance kinetics of the buffer were also monitored under similar conditions. The absorbance of the buffer was subtracted from the absorbance of the proteins at 1,180 s to calculate the subtracted absorbance (Δ*A*_350_). Experiments were performed twice independently in duplicates.

### ANS fluorescence

The fluorescence of 1-anilinonaphthalene-8-sulfonic acid (ANS), purchased from Sigma, bound to ComE and its variants was measured to identify conformational changes in proteins that result in different hydrophobic surfaces. Purified ComE proteins (4 µM) were incubated in buffer containing 20 mM Tris (pH 7.4), 50 mM KCl, and 5 mM DTT for 1 h on ice. Subsequently, reduced proteins were incubated in the presence or absence of 10 mM MgCl_2_ at room temperature. After 20 min, 50 µM ANS was added to each reaction mixture and further incubated for 10 min at room temperature. The reaction mixture was transferred to a multiplate reader (Tecan Spectramax). The fluorescence spectra were measured using an excitation wavelength of *A*_360 nm_ and the emission wavelength range of *A*_400_–*A*_600 nm_. The fluorescence spectra of ANS were also measured. The fluorescence intensities of free ANS were subtracted from the fluorescence intensity of the protein-ANS complex at *A*_500 nm_ to calculate subtracted fluorescence (Δ*F*) (40–42). Experiments were performed twice independently in duplicates.

## RESULTS

### ComE and its homologs activated *nlmA* promoter (P*nlmA*) independent of ComD *in vivo*

**Fig 1:**
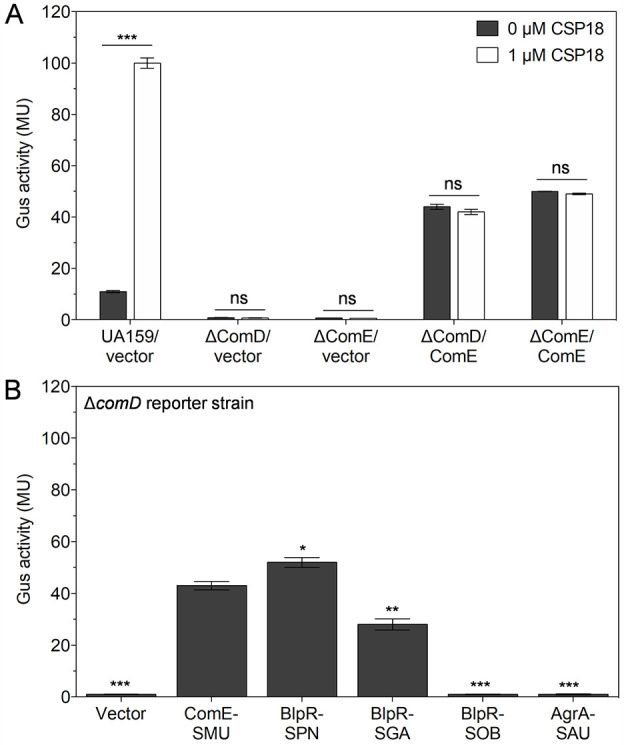
Effects of *S. mutans* ComE and its homolog on the induction of *nlmA* promoter (P*nlmA*) *in vivo* without ComD. (**A**) β-glucuronidase assays of Δ*comE* and Δ*comD* reporter strains containing empty vector pIB184Em or vector with ComE were performed in the absence or presence of 1 µM CSP18 as described in the text. The wild-type UA159 reporter strain transformed with the empty vector was also tested for Gus activity as a control. Gus activity was measured in Miller units (MU). Error bars indicate means ± standard deviation (SD) from four replicates. Statistical analysis was performed using the unpaired *t*-test using GraphPad. (***P < 0.001 and ns, not significant). (**B**) The Gus activity of Δ*comD* reporter strains containing *S. mutans* ComE (ComE-SMU) or its homologs (BlpR from *S. pneumoniae*—BlpR-SPN; BlpR from *S. gallolyticus*—BlpR-SGA; BlpR from *S. sobrinus*—BlpR-SOB; and AgrA from *S. aureus*—AgrA-SAU). Experiments were performed in quadruplicate. Error bars indicate means ± SD. Statistical analysis was performed using the unpaired *t*-test (*P < 0.05, **P < 0.01, and ***P < 0.001).

In general, TCS utilizes a phosphorylation-mediated mechanism to regulate the expression of its target genes. In our previous study, we showed that purified ComE and its cysteine variants could interact with P*nlmA* without phosphorylation (33). In this study, we aimed to investigate the interaction of ComE with cognate promoter in the absence of ComD *in vivo*. To this end, we first constructed a clean *comD-*deleted strain (Δ*comD*) of *S. mutans* UA159, which had native comE and its promoter. The transcriptional fusion P*nlmA-gusA*, prepared in a previous study (33), was integrated at an ectopic location on the Δ*comD* strain to generate a reporter strain IBS1C61 (Table 1). Previously constructed pIB1B68 (pIB184Em containing *S. mutans* ComE with a constitutive promoter P23) or empty vector pIB184Em was transformed into IBS1C61. We used previously constructed strains IBS1C14 (wild-type UA159 reporter strain containing pIB184Em; UA159/vector), IBS1C18 (Δ*comE* reporter strain containing pIB1B68; Δ*comE*/*comE*), and IBS1C20 (Δ*comE* reporter strain containing pIB184Em Δ*comE/*vector), which had P*nlmA-gusA* at the same ectopic location (33). The Gus assays were performed to evaluate the effect of ComE on P*nlmA* expression in different reporter strains in the absence and presence of competence-stimulating peptide CSP18. As expected, for strain IBS1C14, the P*nlmA* expression increased almost 10 times in the presence of exogenous CSP18 (Fig. 1A, UA159/vector). The Gus activity was not observed in the presence or absence of CSP18 in the Δ*comD* and Δ*comE* reporter strains, containing the empty vector (Fig. 1A, Δ*comD*/vector and Δ*comE*/vector). Surprisingly, a comparable Gus activity was observed in the Δ*comD* reporter strain containing pIB184Em carrying wild-type ComE (Δ*comD/comE*) (Fig. 1A, Δ*comD/comE*). When we used the Δ*comE* reporter strain containing pIB184Em with ComE (Δ*comE/comE*), the Gus activity was observed as expected (Fig. 1A, Δ*comE/*ComE). In our previous study, we found that the cysteine variants (C200S and C200S/C229S) of ComE were able to activate P*nlmA* less efficiently than the wild-type ComE in the Δ*comE* reporter strain. Therefore, we checked the Gus activity of these cysteine variants in the Δ*comD* reporter strain and found that these cysteine variants activated P*nlmA* to a similar extent as observed previously in the Δ*comE* reporter strain (Fig. S1A). Overall, these results suggest that the cognate histidine kinase ComD is not required for P*nlmA* activation when ComE is expressed constitutively in *S. mutans* cells. We also noticed that CSP18 did not affect the Gus activity of reporter strains Δ*comE/comE* (Fig. 1A), which could be due to the absence of *comE* at the native location on its chromosome.

In our previous study, we found that *S. mutans* ComE (ComE-SMU) homologs from *S. pneumoniae* (BlpR-SPN) and *S. gallolyticus* (BlpR-SGA) were able to induce P*nlmA* activation, while other ComE homologs, such as BlpR from *S. sobrinus* (BlpR-SOB) and AgrA from *S. aureus* (AgrA-SAU), could not activate P*nlmA* (33). We used the previously constructed plasmids containing the ComE homologs (pIB1B37 [BlpR-SPN], pIB1E1 [BlpR-SGA], pIB1E2 [BlpR-SOB], and pIB1B99 [AgrA-SAU]) (33). These plasmids were transformed into the Δ*comD* reporter strain, and their ability to activate P*nlmA* independently was evaluated. We found that both BlpR-SPN and BlpR-SGA were able to efficiently induce P*nlmA* activation in the absence of ComD (Fig. 1B), suggesting their ability to make functional homodimers with native ComE. The BlpR-SPN activated P*nlmA* slightly higher than the native ComE-SMU, while the activity of BlpR-SGA was slightly lower than the native ComE-SMU (Fig. 1B). Other homologs such as BlpR-SOB and AgrA-SAU did not activate P*nlmA,* suggesting the formation of a non-functional heterodimer. These results were consistent with our previous finding in the Δ*comE* reporter strain. These results suggest that *S. mutans* ComE and some of its homologs can efficiently induce P*nlmA* expression even in the absence of the ComD histidine kinase.

### ComE induced P*nlmA* independent of its phosphorylation status

**Fig 2:**
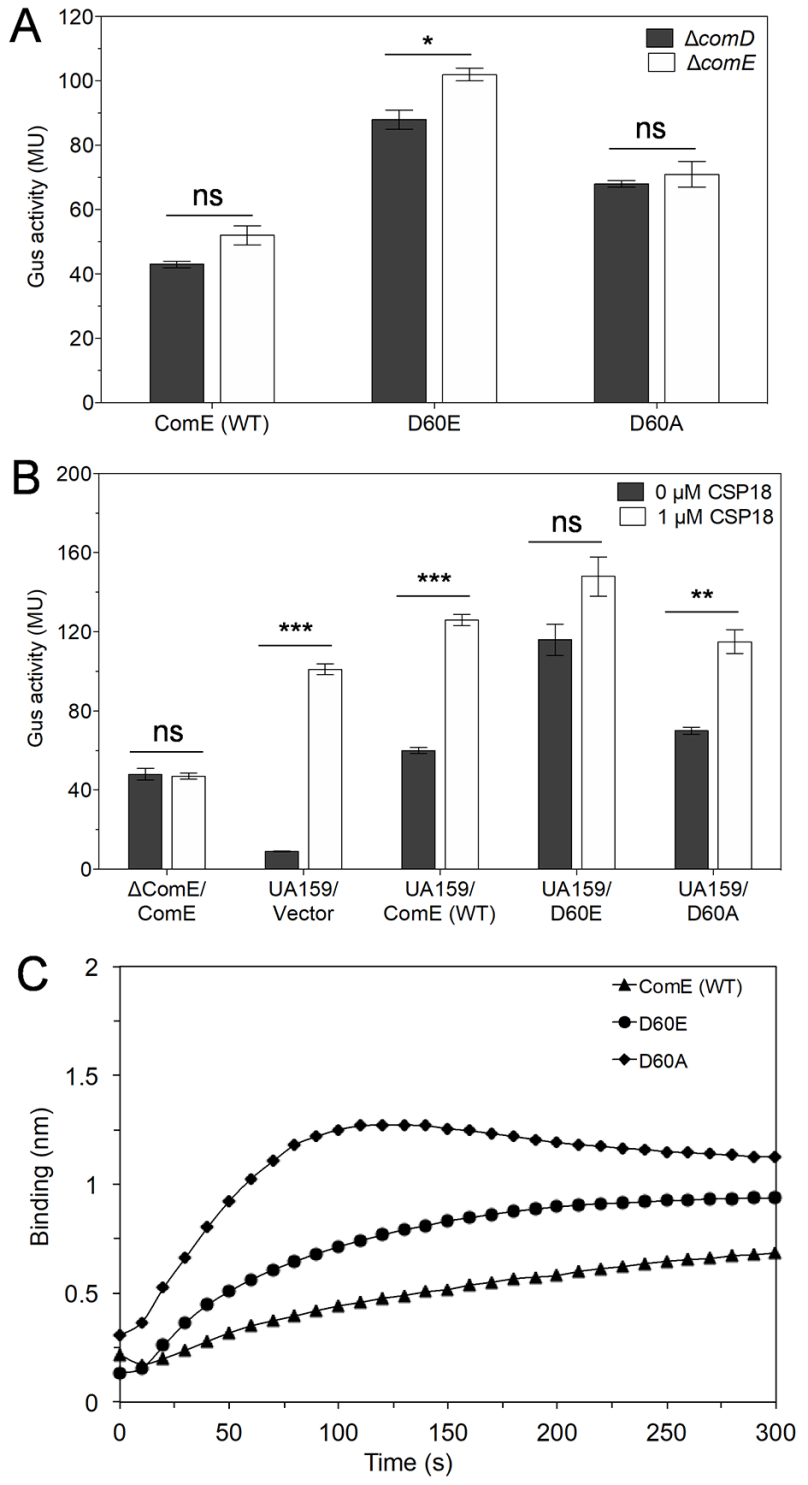
Effects of ComE and its phosphovariants on the P*nlmA* activation. (**A**) The Gus assays of Δ*comE* and Δ*comD* reporter strains supplemented with the phospho-mimicking variant D60E or the non-phosphorylating variant D60A were performed as described above. Experiments were performed in quadruplicate. Error bars indicate means ± standard deviation (SD). Statistical analysis was performed using the unpaired *t*-test (*P < 0.05 and ns, not significant). (**B**) Gus assays of UA159 reporter strains containing empty vector pIB184Em or vector with wild-type ComE (WT) or phospho-mimicking variant or non-phosphorylating variant were performed in the absence or presence of 1 µM CSP18 as described in the text. Experiments were performed two separate times in triplicate. Error bars indicate means ± SD. Statistical analysis was performed using the unpaired *t*-test (**P < 0.01, ***P < 0.001, and ns, not significant). (**C**) The binding of purified ComE (WT) or phospho-mimicking variant D60E or non-phosphorylating variant D60A with P*nlmA* was observed using BLI. A representative graph from three independent sets is shown; the *Y*-axis represents the binding signal in nanometers, and the *X*-axis represents time (s).

Our *in vivo* experiments showed that *S. mutans* ComE and a few homologs could interact with P*nlmA* and activate it for bacteriocin production in the absence of the histidine kinase ComD. This prompted us to investigate further the phosphorylation-independent role of ComE in *S. mutans*. In previous studies, the response regulators are shown to be phosphorylated on a conserved aspartate residue (43–45). A change in aspartate residue to glutamate in some response regulators is known to mimic the phosphorylated form of the response regulator (46). Although Hung and colleagues (45) have shown that Asp 60 (D60) residue of *S. mutans* ComE is the phosphorylation site, their study lacked the *in vivo* demonstration and validation of the D60 residue. We therefore constructed phospho-mimicking variant (D60E) and phospho-inactivating variant (D60A) and named pIB1B98 and pIB1B96, respectively. These plasmids, including pIB1B68 (ComE [WT]), were introduced into the Δ*comD* and Δ*comE* reporter strains, and the Gus activities were determined. As shown in Fig. 2A, in both the reporter strains, Δ*comD* and Δ*comE,* containing the wild-type ComE (WT), Gus activity was observed. As expected, the phospho-mimicking variant D60E showed nearly twice the Gus activity compared to the ComE (WT), suggesting that the D60E variant activates P*nlmA* (Fig. 2A, D60E). Surprisingly, the non-phosphorylating variant, D60A, also showed Gus activity, indicating that it also activates P*nlmA* expression (Fig. 2A, D60A). We then constructed another non-phosphorylating variant, D60N. We found that the D60N variant also induced P*nlmA* (Fig. S1).

It is possible that aspartate or glutamate residues present in the nearby region of the D60 residue of ComE are the actual residues that are phosphorylated during signal transduction. Therefore, we analyzed the *S. mutans* ComE amino acid sequence and found four glutamate residues located at the 62nd, 66th, 67th, and 72nd positions of ComE. We then constructed a double ComE variant where, in addition to D60A, glutamate at position 66 was replaced with alanine, D60A/E66A. We also constructed a ComE variant where, in addition to D60A, all the glutamate residues were replaced with an alanine residue (D60A/E62A/E66A/E67A/E72A). These ComE variants were introduced into Δ*comD* and Δ*comE* reporter strains and evaluated for their ability to induce P*nlmA*. We found that both the variants D60A/E66A and D60A/E62A/E66A/E67A/E72A induced P*nlmA* expression in both the reporter strains (Fig. S1B). Thus, the possibility of involvement of nearby aspartate or glutamate residues in phosphorylation was nullified. These results also indicate that, although ComE phosphorylation enhances the transcriptional activation of P*nlmA*, unphosphorylated ComE is also able to induce P*nlmA* expression when constitutively expressed and present in higher copy number.

Next, we were interested in exploring the co-expression effect of native ComE and the supplemented wild-type ComE and its phosphovariants on the P*nlmA* activation. We introduced the WT ComE and phosphovariant ComE (pIB1B98 and pIB1B96) constructs into the UA159 reporter strain. We measured the PnlmA activation in the presence and absence of additional CSP18. As mentioned above, the Gus activity of the Δ*comE*/*comE* and UA159/vector reporter strains was also observed. As expected, the Δ*comE*/*comE* reporter strain activated the P*nlmA* expression independent of CSP18 addition (Fig. 2B). However, we observed that the Gus activity of the reporter strain UA159/vector increased nearly 10 times higher when CSP18 was added, suggesting CSP-dependent activation of the P*nlmA* promoter (Fig. 2B). However, when wild-type ComE is expressed in the UA159 reporter strain (UA159/ComE [WT]), the basal level of Gus activity without CSP18 was similar to the Gus activity of the Δ*comE*/*comE* reporter strain. Furthermore, we found that without the addition of CSP18 to the UA159 reporter strain (UA159/ComE [WT]), the Gus activity was nearly six times higher compared to the UA159/vector reporter strain. Interestingly, the Gus activity of the UA159/ComE (WT) reporter strain was also higher than that of the Δ*comE*/*comE* reporter strain when CSP18 was present, suggesting a cumulative effect of native ComE and supplemented ComE on P*nlmA* expression. The non-phosphorylating variant D60A also showed an increase in Gus activity when native ComE is expressed (Fig. 2B). As expected, the phospho-mimicking variant D60E did not show a significant increase in Gus activity when CSP18 was added. Cumulatively, these results indicate that although phosphorylation of ComE is mediated by ComD when activated by CSP18, non-phosphorylating ComE is capable of inducing P*nlmA* expression.

To investigate the phosphorylation-dependent or independent interaction of ComE with P*nlmA in vitro*, ComE (WT), D60E, and D60A constructs with 6× histidine-tag were prepared in pET15b vector, and proteins were isolated from *E. coli* BL21 (DE3) cells as described in Materials and Methods. In our previous study, we showed that purified ComE forms dimers/oligomers using intra- and inter-molecular disulfide bonds (33). We checked the dimerization/oligomerization ability of both variants D60E and D60A on SDS-PAGE. As shown in Fig. S2A, both the variants formed dimers/oligomers similar to the wild-type ComE protein under non-reducing conditions (33). However, for this study, we used reducing conditions for *in vitro* experiments. The binding association rate constants of ComE (WT) and its variants to P*nlmA* were calculated using BLI. The association rate constants for ComE (WT), D60E, and D60A to P*nlmA* were found to be 1.1 ± 0.1 × 10^4^, 1.8 ± 0.2 × 10^4^, and 2.8 ± 0.4 × 10^4^ M^−1^ s^−1^, respectively. As expected, the phospho-mimicking variant, D60E, showed almost 1.5 times higher association rate than ComE (WT), suggesting that D60E is capable of binding to P*nlmA* (Fig. 2C). Surprisingly, we also found that the non-phosphorylating variant D60A could strongly interact with P*nlmA*. These results support our *in vivo* findings that, although phosphorylation of ComE leads to strong binding with its target promoter, ComE can efficiently bind to its promoter without phosphorylation.

### D60 residue of ComE is required for its phosphorylation *in vitro*

**Fig 3:**
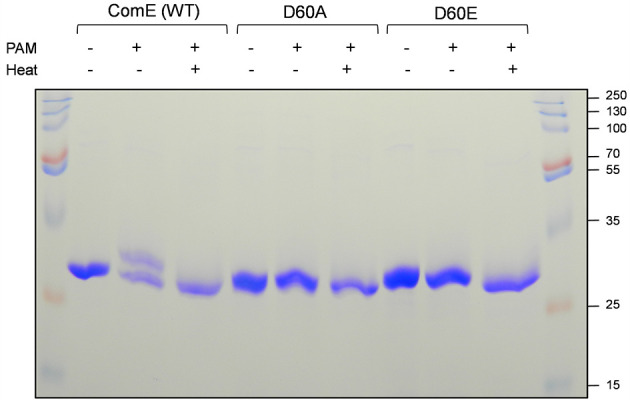
Importance of D60 residue of ComE for phosphorylation. The phosphorylation ability of purified ComE (WT) or phosphovariants (D60A and D60E) was tested using phosphoramidate on Phos-tag SDS-PAGE. The proteins were processed for phosphorylation as mentioned in the text. Samples were heated at 95°C for 5 min whenever mentioned. Samples were loaded on 12% Phos-tag SDS-PAGE and run at 90 V for 4 h. Experiments were performed at least twice, and a representative gel is shown.

We found that the non-phosphorylating variant of ComE, D60A, is able to interact with P*nlmA* and induce its activation. It is possible that ComE is phosphorylated at aspartate residues other than at the 60th position. Therefore, we desired to confirm whether the D60 residue of ComE is only responsible for the phosphorylation during signal transduction. Response regulators can be phosphorylated *in vitro* by high-energy phosphate donors such as phosphoramidate (PAM) and acetyl phosphate *in vitro* (47, 48) (16). We tested the phosphorylation of ComE (WT), D60A, and D60E with PAM using Phos-tag SDS-PAGE (Fig. 3). Phos-tag gel is used to separate phosphorylated and non-phosphorylated proteins (49). Phosphorylated proteins move more slowly than non-phosphorylated proteins. The purified proteins were subjected to phosphorylation using PAM as described in Materials and Methods. As shown in Fig. 3, we observed a predominant ~31 kDa band when ComE (WT) was not treated with PAM, which is the non-phosphorylated form of ComE. When PAM was added, we observed two bands on the Phos-tag gel: one band at 31 kDa size that corresponds to the non-phosphorylated form and a slower migrating band that is higher than 31 kDa in size, which we believe is the phosphorylated form. When we heat treated the sample, the slow-migrating species disappeared, confirming that it was the phosphorylated form of ComE. When we treated the ComE variants D60A and D60E with PAM, the phosphorylated form was not observed. These results indicate a high possibility that the D60 residue of ComE is responsible for phosphorylation during signal transduction. We also attempted to detect ComE (WT) phosphorylation using acetyl phosphate, as CovR was shown previously to be phosphorylated with acetyl phosphate (16). Although the phosphorylated form of CovR was observed, the acetyl phosphate could not phosphorylate ComE (WT) in non-reducing conditions or reducing conditions (Fig. S2B). These results suggest that PAM is a better phosphorylating agent for ComE than acetyl phosphate.

### Mg^2+^ triggered the assembly of ComE

**Fig 4:**
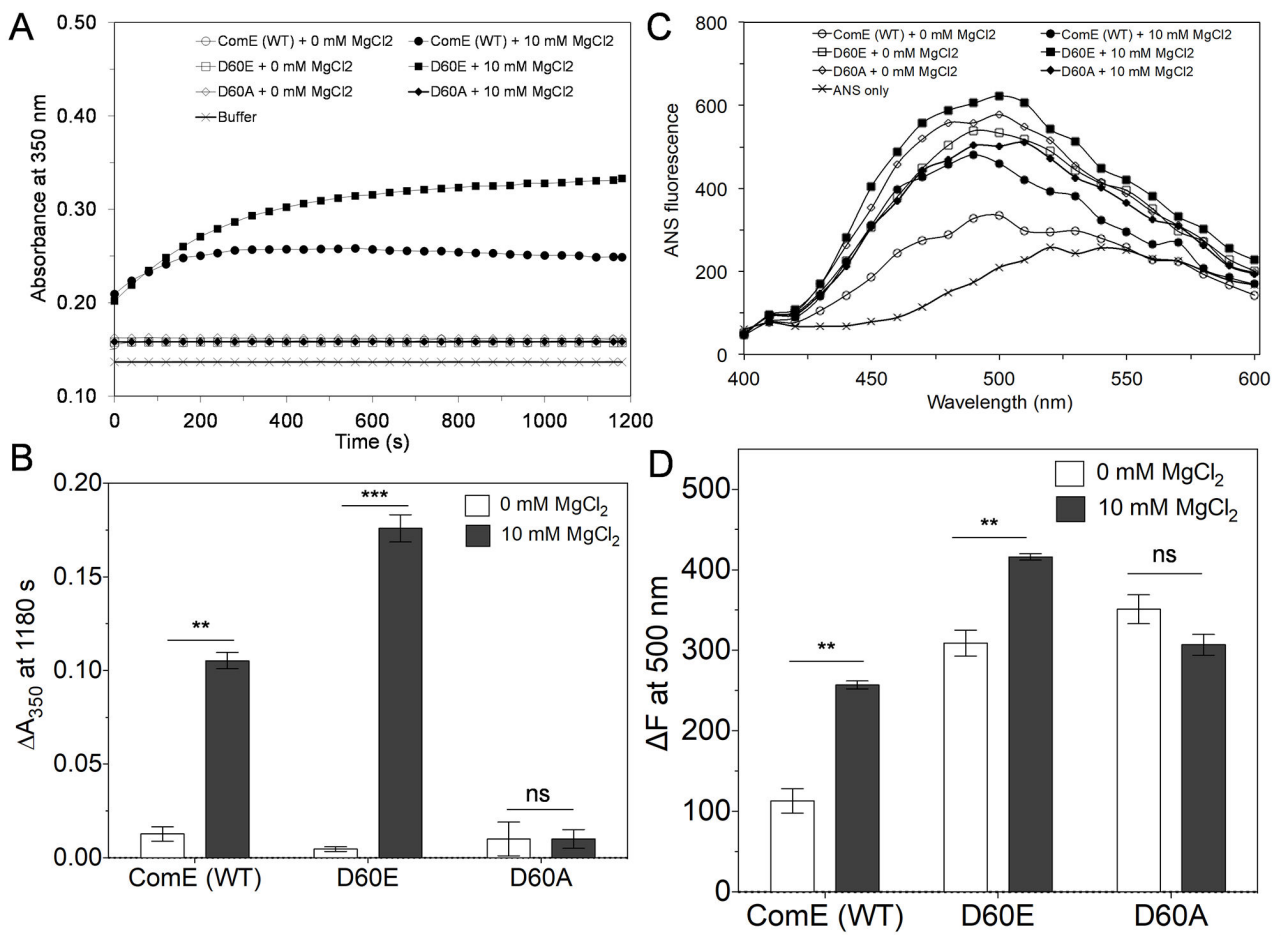
Effects of magnesium chloride on the assembly of ComE and its phosphovariants. (**A**) The effect of magnesium chloride (MgCl_2_) was observed on the assembly of ComE (WT) or phosphovariants (D60E and D60A) by absorbance kinetics. Purified proteins (4 µM) were incubated in a buffer containing 20 mM Tris (pH 7.4), 50 mM KCl, and 5 mM DTT at 4°C for 1 h. The absorbance at 350 nm was monitored for ~20 min at room temperature in the absence or presence of 10 mM MgCl_2_. (**B**) The absorbance of buffer at 350 nm after 20 min (1,180 s) was subtracted from the absorbance of different protein samples to calculate the subtracted absorbance at 350 nm (Δ*A*_350_). The Δ*A*_350_ of proteins was plotted in the absence and presence of 10 mM MgCl_2_. Experiment was performed in triplicate. Error bars indicate means ± standard deviation (SD). Statistical analysis was performed using the unpaired *t*-test (**P < 0.01, ***P < 0.001, and ns, not significant]. (**C**) The conformation of purified ComE (WT) and phosphovariants (D60A and D60E) was observed using ANS fluorescence. Proteins (4 µM) were incubated as described above. Then, reactions were incubated at room temperature for 20 min without and with 10 mM MgCl_2_, followed by 5 min incubation with 50 µM ANS. The fluorescence intensities of ANS only and ANS bound with proteins were observed using excitation wavelength (360 nm) and emission wavelength (400–600 nm). (**D**) The fluorescence intensities of ANS at 500 nm were subtracted from the fluorescence intensities of ANS-bound proteins to calculate subtracted fluorescence (Δ*F*). The Δ*F* at 500 nm of these proteins was plotted without and with 10 mM MgCl_2_. Experiments were performed two independent times in duplicates. Error bars indicate means ± SD. Statistical analysis was performed using the unpaired *t*-test (**P < 0.01 and ns, not significant).

Divalent cations such as magnesium are known to trigger self-assembly of proteins (50). Some response regulators, including *S. mutans* ComE, are known to form oligomers spontaneously (45, 51, 52). In our previous study, we found that ComE is able to form cysteine-mediated dimers and oligomers (33). Thus, we wanted to verify whether MgCl_2_ could affect the assembly of ComE and its phosphovariants. We examined the turbidity of ComE and its variants in the absence and presence of MgCl_2_ by detecting absorbance at 350 nm (*A*_350_). This method has been used previously to observe the protein assembly, which results in the formation of larger structures or aggregates (37–39). Without MgCl_2_, the absorbance at *A*_350_ of ComE (WT) and its variants (D60E, D60A) did not increase with time, and the absorbance kinetics were similar to the absorbance kinetics of the buffer, suggesting no assembly formation (Fig. 4A). Surprisingly, we found that in the presence of MgCl_2_, the absorbance at *A*_350_ of ComE (WT) and D60E increased with time; however, the absorbance of the D60A variant remained the same (Fig. 4A). We then subtracted the *A*_350_ value of the buffer from the *A*_350_ values of all the proteins to obtain a subtracted absorbance value (Δ*A*_350_). As shown in Fig. 4B, in the absence of MgCl_2_, the Δ*A*_350_ values of ComE (WT), D60E, and D60A were negligible at 0.01, 0.01, and 0.01, respectively (Fig. 4B). In contrast, when MgCl_2_ was present, the Δ*A*_350_ values of ComE (WT) and D60E increased to 0.1 and 0.2, respectively. On the other hand, the Δ*A*_350_ value of D60A remained unchanged at 0.01 (Fig. 4B). These results suggested that both ComE (WT) and D60E proteins had the ability to self-assemble, but D60A could not self-assemble.

It is well known that a single point mutation in a protein can significantly affect the integrity of the tertiary structure and its function (53–55). ANS is known to bind with hydrophobic surfaces of proteins, and upon binding to proteins, the fluorescence of the protein-ANS complex increases significantly compared to the free ANS (40–42). In our recent study, we found that ANS fluorescence of a double cysteine variant of ComE was 10–15 times higher than ComE (WT), indicating a much wider conformational change (33). We also noticed that the ANS fluorescence of ComE dimers/oligomers was significantly higher than the monomers. This could be due to the increase in the hydrophobic surfaces during the dimerization/oligomerization process. Thus, we thought to investigate whether D60 mutations in ComE could perturb its conformation and whether ANS fluorescence changes during self-assembly. The purified ComE (WT), D60E, and D60A proteins were incubated in the presence or absence of 10 mM MgCl_2_, followed by the addition of 50 µM ANS, and the ANS fluorescence spectra were measured. As shown in Fig. 4C, the fluorescence of ComE (WT) and its variants was increased ~2–3 times when incubated with ANS. The fluorescence maxima of ANS were also shifted from 540 nm (free ANS) to 500 nm (ANS-protein complex), suggesting a strong binding of ANS to ComE proteins. We then subtracted the fluorescence intensity of ANS at 500 nm from the fluorescence intensities of the ANS-protein complexes to calculate the subtracted fluorescence value (Δ*F*). We found that in the absence of MgCl_2_, the Δ*F* values of ComE (WT), D60E, and D60A were 113 ± 15, 309 ± 16, and 351 ± 18, respectively. Notably, the Δ*F* values of D60E and D60A were ~3 times higher than the ComE (WT) protein, suggesting that these variants have higher hydrophobic surfaces. The Δ*F* value of D60E was similar to D60A, suggesting that both variants have similar hydrophobic surfaces. These results suggest that mutations at the D60 residue of ComE significantly affected the tertiary structure. However, in the presence of MgCl_2_, the Δ*F* value of ComE (WT) and D60E increased to 257 ± 5 and 416 ± 4, respectively. On the other hand, the Δ*F* value of D60A (307 ± 13) remained similar to the Δ*F* value when MgCl_2_ was absent. Collectively, these results indicated that ComE (WT) and D60E had higher hydrophobic surfaces, likely due to their assembly in the presence of MgCl_2_, while hydrophobic pockets of D60A remained similar in the presence or absence of MgCl_2_.

### *In vivo* heteromeric ComE activated P*nlmA* weakly than its homomers

Response regulators are known to form dimers/oligomers for the activities during signal transduction (56–60). *S. mutans* ComE has also been shown to form oligomers in previous *in vitro* studies (45). Therefore, we wanted to check whether native ComE has the ability to interact with its variants and homologs *in vivo*. As mentioned before (Fig. 1B), we found that ComE homologs such as BlpR-SPN and BlpR-SGA could activate P*nlmA,* and hence, they were functional in *S. mutans*. While other homologs, such as BlpR-SOB and AgrA-SAU, were non-functional in *S. mutans*.

**Fig 5:**
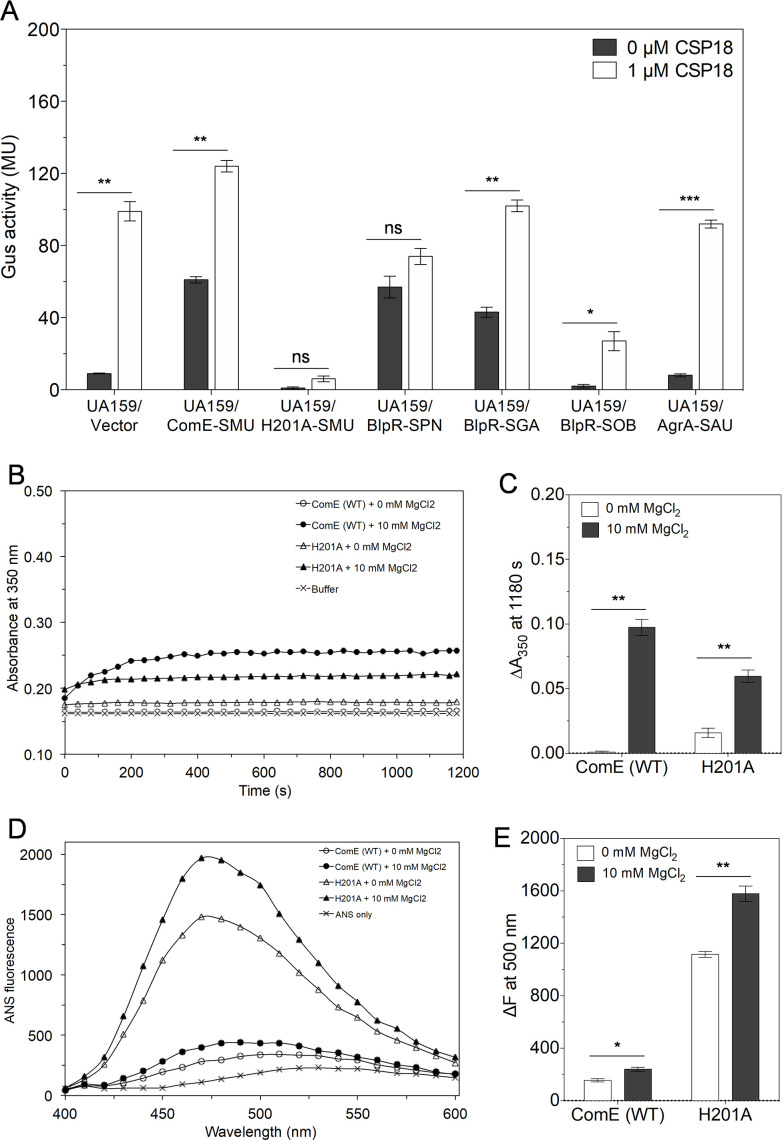
Functional evaluation and conformational changes of ComE variants. (**A**) Gus assays of UA159 reporter strains containing empty vector (pIB184Em) or vector carrying ComE-SMU or ComE H201A-SMU or and its homologs BlpR-SPN, BlpR-SGA, BlpR-SOB, and AgrA-SAU were performed in the absence or presence of 1 µM CSP18. Experiments were performed two independent times in triplicate. Error bars indicate means ± standard deviation (SD). Statistical analysis was performed using the unpaired *t*-test (*P < 0.05, **P < 0.01, ***P < 0.001, and not significant). (**B**) The absorbance kinetics of ComE (WT) or ComE H201A were monitored for 20 min (1,180 s) under similar conditions as described above. (**C**) The Δ*A*_350_ of both proteins at 20 min was plotted in the absence and presence of 10 mM MgCl_2_. Experiment was performed two times independently in duplicates. Error bars indicate means ± SD. Statistical analysis was performed using the unpaired *t*-test (**P < 0.01). (**D**) The ANS fluorescence spectra of ComE (WT) or ComE H201A were recorded under similar conditions as described above. (**E**) The Δ*F* at 500 nm of both proteins was plotted in the absence and presence of 10 mM MgCl_2_. Experiment was performed two times independently in duplicates. Error bars indicate means ± SD. Statistical analysis was performed using the unpaired *t*-test (*P < 0.05 and **P < 0.01).

To investigate further, we measured the Gus activity of the ComE (WT), ComE variant (H201A), and the ComE homologs (BlpR-SPN, BlpR-SGA, BlpR-SOB, and AgrA-SAU) in the UA159 reporter strain in the presence or absence of CSP18. The UA159 reporter strain supplemented with empty vector showed Gus activity of 10, which increased to 99 ± 5 when CSP18 was added. When this reporter strain was supplemented with ComE (WT), the Gus activity was 61 ± 2 in the absence of CSP18, which increased to 111 ± 4 after the addition of CSP18. The change in the Gus values for both strains was similar to the changes described above. As noted above, the reporter strain supplemented with ComE (WT) exhibited higher Gus activity than the strain with the vector control, which we believe was due to the higher expression of ComE (WT) from the plasmid (Fig. 5A). In contrast, the reporter strain containing a nonfunctional variant of ComE, H201A, showed no measurable Gus activity (Fig. 5A). With the addition of CSP18, the Gus activity of this variant was only 6 ± 2, which is ~18 times lower than the reporter strain with ComE (WT). Thus, it is possible that H201A forms a heterodimer with ComE (WT), which is nonfunctional. The reporter strains containing BlpR-SGA and BlpR-SPN homologs showed Gus activity of 43 ± 3 and 57 ± 6, respectively. With CSP18 addition, the Gus activity increased to 102 ± 3 for BlpR-SGA. However, for BlpR-SPN, the Gus activity (74 ± 5) did not increase significantly upon CSP18 addition. The reporter strain containing the nonfunctional homolog BlpR-SOB showed very little activity. However, upon the addition of CSP18, Gus activity increased to 27 ± 5, which was four times lower than that of ComE (WT). For the reporter strain supplemented with AgrA of *S. aureus,* the Gus activity was 8 even without CSP18. With CSP18 addition, the activity increased to 92 ± 2, a value similar to the reporter strain carrying an empty vector. This suggests that AgrA did not impact ComE function.

We next measured the absorbance kinetics of ComE (WT) and the nonfunctional variant H201A to evaluate the assembly ability of H201A (17). We found that the *A*_350_ values of ComE (WT) and H201A increased with time, indicating that H201A could assemble. As shown in Fig. 5C, the Δ*A*_350_ of H201A was ~1.5 times less than that of ComE (WT), suggesting reduced assembly. Furthermore, the ANS fluorescence of ComE (WT) and H201A was measured to check whether this nonfunctional variant has a different conformation. The fluorescence intensities of both ComE (WT) and H201A were increased when bound to ANS, and blue shifts were observed, suggesting strong ANS binding. The subtracted fluorescence of H201A was seven times higher than ComE (WT), suggesting much wider hydrophobic surfaces in the variant. With the addition of MgCl_2_, as expected, the subtracted fluorescence intensities of both proteins increased. These results suggest that the nonfunctional variant H201A demonstrates a different conformation with open hydrophobic pockets compared to ComE (WT). The conformational difference between ComE (WT) and H201A was verified using their digestion with proteases chymotrypsin, trypsin, and proteinase K on SDS-PAGE. The banding patterns of cleaved ComE (WT) and H201A proteins were similar in the presence of each protease, suggesting that these proteases cleaved both proteins similarly (Fig. S2C). Since the ANS fluorescence study showed that H201A had an open conformation, we tested its ability to be phosphorylated by PAM. We found that H201A was phosphorylated by PAM, suggesting that the wider conformation did not interfere with the phosphorylation of protein (Fig. S2D).

## DISCUSSION

Among the ~14 prototypical TCS systems encoded by the genome of various *S. mutans* strains*,* the ComDE system is one of the most important systems that has been extensively studied. This system is predominantly involved in the regulation of bacteriocin production, and it shares the highest homology with the bacteriocin-regulating two-component system BlpRH of *S. pneumoniae*. Although this system was initially studied for other cellular processes, including biofilm formation and competence development (31, 61–64) and named as ComDE, it was recently renamed as BlpRH (65) due to its primary function in both lantibiotic (SmbAB) and nonlantibiotic (NlmAB) bacteriocin production. However, in this communication, we have decided to use ComDE nomenclature to be consistent with our previous study (33).

This TCS system is activated in a cell-density-dependent manner that is reliant on the quorum-sensing peptide, CSP, which is encoded by the TCS operon. The primary function of the ComDE system in *S. mutans* UA159 is to activate NlmAB bacteriocin expression and secretion. In fact, by random mutagenesis screening, we have originally identified all the genes in the regulatory circuit of the bacteriocin NlmAB production—ComC, ComD, ComE, SepM, and NlmTE, along with global regulators like SprV (66).

**Fig 6:**
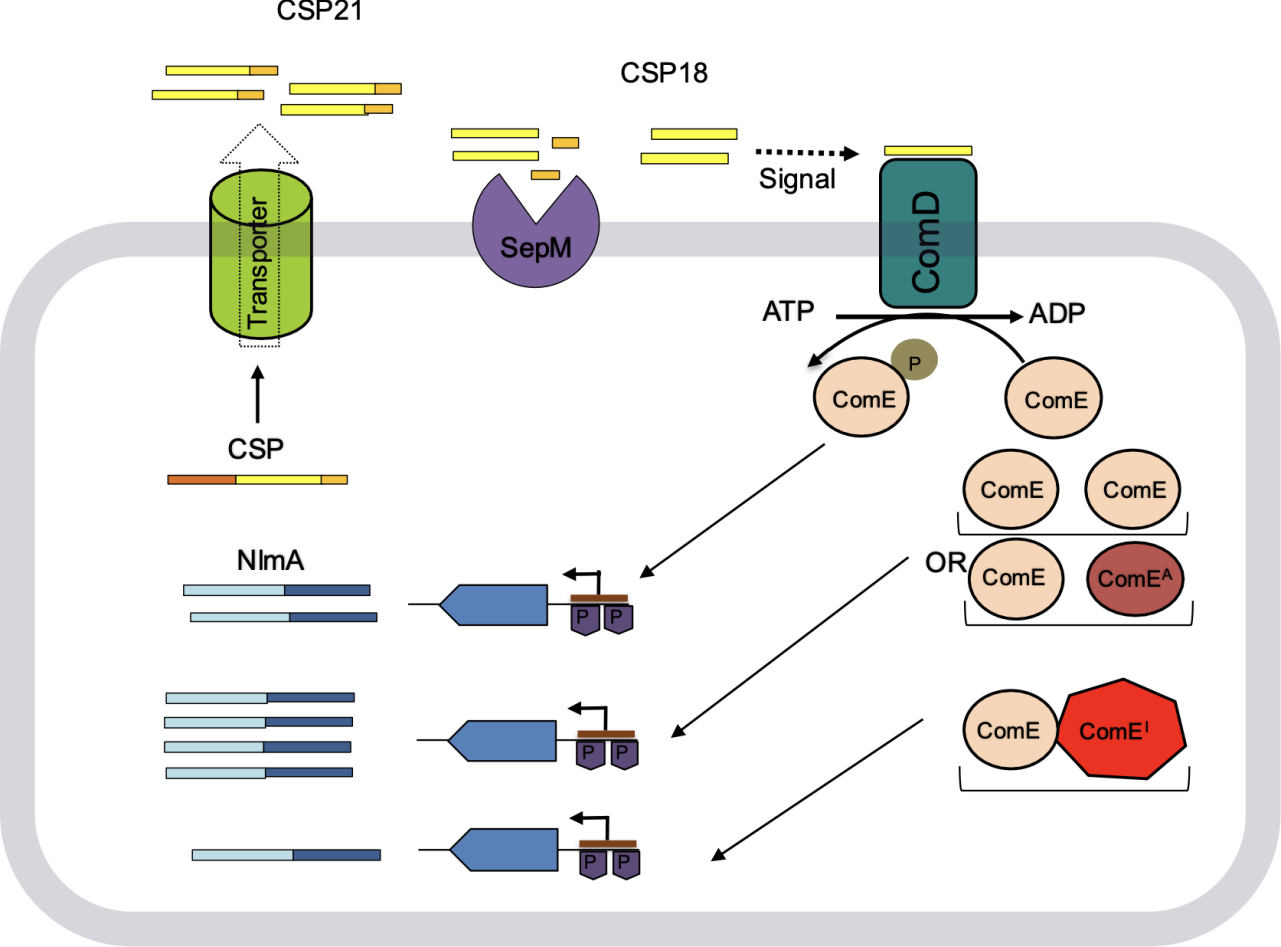
A model for the regulation of the ComDE pathway in *S. mutans* is shown. The two-component system ComDE consists of a membrane-bound histidine kinase ComD, a cytoplasmic response regulator ComE, and a competence-stimulating peptide CSP. The ComD interacts with the mature form of signal peptide CSP18 and activates and catalyzes ATP for its autophosphorylation. The phosphorylated ComD interacts with cognate response regulators ComE, leading to its phosphorylation (P). The phosphorylated ComE binds to the cognate promoters and regulates gene expression. Due to physiological changes or self-assembly, if ComE concentration becomes high in the cell, ComE can then interact with the cognate promoters even without its phosphorylation. Similarly, phosphorylation is not required for *S. mutans* ComE homologs BlpR-SPN and BlpR-SGA, which could accomplish the function of ComE in the absence of ComD. These active BlpRs are shown as ComE^A^. The other ComE homologs, such as BlpR-SOB and ComE variant H201A, are unable to activate transcription in the absence or presence of ComD and are shown as ComE^I^. These heterologous ComE^I^ are able to interact with innate ComE in the cell, form heterodimers, and only activate the promoter weakly. The TCS regulates several gene products required for cellular responses. Small peptide CSP is one of the products of these cellular responses, which triggers this process. These peptides are cleaved into a 21 amino acid peptide (CSP21) and exported to the extracellular environment through a transporter system. The peptide CSP21 is further cleaved by the protease SepM to generate a mature form named CSP18. The CSP18 acts as a signal for histidine kinase ComD.

In this study, we made two unprecedented observations regarding the ComDE system. First, we found that at a higher concentration, ComE can activate its target gene expression even in the absence of its cognate sensor kinase, ComD (Fig. 1). Second, we observed that at higher concentrations, the phosphorylation of the conserved aspartate residue (D60) in ComE is not required for target gene expression (Fig. 2), as illustrated in the model in Fig. 6. While the literature provides several examples of orphan response regulators that function without their cognate sensor kinases, our findings suggest a unique mechanism. For instance, CovR, an orphan regulator in *S. mutans*, is a part of the well-known CovRS system identified in *Streptococcus pyogenes* (67–71). Similarly, RitR in *S. pneumoniae*, which regulates iron transport, is an orphan regulator (72). Other examples of orphan response regulators include Aor1, DegU, DigR, EpsW, and Hp1043 found in both gram-positive and gram-negative bacteria (73–77). These regulators often rely on non-cognate sensor kinases through a mechanism known as crosstalk (13, 78–81). In contrast, we propose that ComE activation in the absence of ComD occurs through a mechanism independent of such crosstalk.

To this end, Reck and colleagues (82) have observed that BlpR-dependent gene activation occurs in a BlpH (sensor kinase)-deficient strain, suggesting that either BlpR receives input from a non-cognate HK (probably ComD), or the non-phosphorylated form is involved in gene activation (82, 83). Both BlpH and ComD are HPK10 family sensor kinases. They contain conserved motifs such as H-box (IRSFRHDYXNILTSLR), N-box (LXDNAIEAA), and G-box (STKGXXRGIGLA). H-box, N-box, and G-box are essential for autophosphorylation and ATP binding (84, 85). Streptococci that belong to the mitis and anginosus groups encode two types of HPK10 subfamilies, while other streptococci encode only one (65). However, it is very uncommon for sensor kinases to crosstalk with other response regulators, especially when the signaling molecule is a peptide (12, 86).

Incidentally, we also found that phosphorylation of ComE is not necessary for gene activation since at higher concentrations, both the D60A and D60N mutants behaved similarly to the wild-type ComE (Fig. 2 and 6; Fig. S1B). While the dispensable nature of aspartate phosphorylation of response regulator seems incongruous, there are several instances where phosphorylation is not necessary. For example, in the ArsRS TCS system of *Helicobacter pylori*, it has been shown that the response regulator ArsR can modulate gene expression in a phosphorylation-independent manner (87, 88).

It is important to mention that we also observed the phospho-mimetic mutant D60E activated gene expression nearly twice as compared to the wild-type ComE, suggesting that phosphorylation positively influences ComE activity (Fig. 4). Furthermore, we also found that D60E undergoes a larger shift as compared to the wild-type ComE in the presence of MgCl_2_, which may indicate that D60E multimerizes better compared to the wild-type ComE. A combination of experiments, such as dynamic light scattering and electron microscopy, is required to confirm whether the observed phenomena are due to a higher degree of polymerization. At this point, the exact mechanisms for this increased gene expression by the D60E variant are not entirely known.

We have previously shown that two ComE homologs, BlpR-SpN and BlpR-SGA, could successfully accomplish ComE function in *S. mutans* (33). However, here we found that at higher concentrations, these two homologs also do not require the histidine kinase ComD for the activity, similar to the *S. mutans* ComE (Fig. 1B). In the presence of wild-type ComD and ComE (UA159) (Fig. 5), we observed that the addition of exogenous CSP overrides the multimerization effect on gene expression by ComE and its homologs. However, we did not evaluate whether some of the ComE homologs were phosphorylated and functional *in vivo*. Therefore, the reported observation (Fig. 1B) that overexpression of unphosphorylated response regulators can trigger activation is likely explained by the fact (Fig. 5A) that these proteins exist in equilibrium between unphosphorylated and phosphorylated conformations. At high exogenous CSP levels, even the small fraction in the wild-type phosphorylated ComE may surpass the threshold required to initiate activation.

The DNA binding domain of AgrA, ComE, and the homologs contains a LytTR motif, which is approximately 100 residues long (89, 90). A recent systematic mutation study of the LytTR domain of AgrA found that several conserved residues in this region, including H201, are needed for biological function (17). Since the conserved histidine residue is close to the cysteine residue (C200) that takes part in disulfide bond formation, we made the H201A replacement. This H201A variant was nonfunctional, consistent with the previous study (17). We also found that this mutant underwent wider conformational changes; however, this conformational change did not interfere with the phosphorylation (Fig. 5; Fig. S2). The differences observed in ANS fluorescence, indicating a conformational change, can be attributed to their distinct working principles, while protease cleavage does not. ANS fluorescence can detect subtle changes in protein conformation that may not significantly affect the protease cleavage sites, indicating that these conformation changes might involve minor shifts or rearrangements that are enough to alter ANS binding but not to expose or alter the accessibility of the protease cleavage sites. As a result, no significant differences are detected. The reason why this mutant is nonfunctional should be studied in the future.

It is important to mention that in this study, we have only evaluated *S. mutans* and no other streptococci. The TCS ComDE (i.e., BlpRH) is highly conserved among streptococci (65). Furthermore, streptococci belonging to the salivarius group contain two or more of this TCS system, where they regulate bacteriocin production (65). Thus, it will be necessary to evaluate whether ComE functions in a similar manner in these streptococci or functions differently than in *S. mutans*. We plan to evaluate this in our future studies.

## SUPPLEMENTAL MATERIAL

Supplemental figures spectrum.01477-25-s0001.pdf Figures S1 and S2.

ASM does not own the copyrights to Supplemental Material that may be linked to, or accessed through, an article. The authors have granted ASM a non-exclusive, world-wide license to publish the Supplemental Material files. Please contact the corresponding author directly for reuse.

## References

[1] Bowden GH, Hamilton IR. 1998. Survival of oral bacteria. Crit Rev Oral Biol Med 9:54–85. doi:10.1177/10454411980090010401 9488248

[2] Burne RA. 1998. Oral streptococci... products of their environment. J Dent Res 77:445–452. doi:10.1177/00220345980770030301 9496917

[3] Kolenbrander PE. 2000. Oral microbial communities: biofilms, interactions, and genetic systems. Annu Rev Microbiol 54:413–437. doi:10.1146/annurev.micro.54.1.413 11018133

[4] Kroes I, Lepp PW, Relman DA. 1999. Bacterial diversity within the human subgingival crevice. Proc Natl Acad Sci U S A 96:14547–14552. doi:10.1073/pnas.96.25.14547 10588742 PMC24473

[5] Dewhirst FE, Chen T, Izard J, Paster BJ, Tanner ACR, Yu W-H, Lakshmanan A, Wade WG. 2010. The human oral microbiome. J Bacteriol 192:5002–5017. doi:10.1128/JB.00542-10 20656903 PMC2944498

[6] Evaldson G, Heimdahl A, Kager L, Nord CE. 1982. The normal human anaerobic microflora. Scand J Infect Dis Suppl 35:9–15. 6762655

[7] Paster BJ, Boches SK, Galvin JL, Ericson RE, Lau CN, Levanos VA, Sahasrabudhe A, Dewhirst FE. 2001. Bacterial diversity in human subgingival plaque. J Bacteriol 183:3770–3783. doi:10.1128/JB.183.12.3770-3783.2001 11371542 PMC95255

[8] Kuramitsu HK. 1993. Virulence factors of mutans streptococci: role of molecular genetics. Crit Rev Oral Biol Med 4:159–176. doi:10.1177/10454411930040020201 8435464

[9] Stock AM, Robinson VL, Goudreau PN. 2000. Two-component signal transduction. Annu Rev Biochem 69:183–215. doi:10.1146/annurev.biochem.69.1.183 10966457

[10] Jacob-Dubuisson F, Mechaly A, Betton JM, Antoine R. 2018. Structural insights into the signalling mechanisms of two-component systems. Nat Rev Microbiol 16:585–593. doi:10.1038/s41579-018-0055-7 30008469

[11] Mitrophanov AY, Groisman EA. 2008. Signal integration in bacterial two-component regulatory systems. Genes Dev 22:2601–2611. doi:10.1101/gad.1700308 18832064 PMC2751022

[12] Podgornaia AI, Laub MT. 2013. Determinants of specificity in two-component signal transduction. Curr Opin Microbiol 16:156–162. doi:10.1016/j.mib.2013.01.004 23352354

[13] Capra EJ, Laub MT. 2012. Evolution of two-component signal transduction systems. Annu Rev Microbiol 66:325–347. doi:10.1146/annurev-micro-092611-150039 22746333 PMC4097194

[14] Aiba H, Nakasai F, Mizushima S, Mizuno T. 1989. Phosphorylation of a bacterial activator protein, OmpR, by a protein kinase, EnvZ, results in stimulation of its DNA-binding ability. J Biochem 106:5–7. doi:10.1093/oxfordjournals.jbchem.a122817 2674113

[15] Lejona S, Castelli ME, Cabeza ML, Kenney LJ, García Véscovi E, Soncini FC. 2004. PhoP can activate its target genes in a PhoQ-independent manner. J Bacteriol 186:2476–2480. doi:10.1128/JB.186.8.2476-2480.2004 15060051 PMC412160

[16] Khara P, Mohapatra SS, Biswas I. 2018. Role of CovR phosphorylation in gene transcription in Streptococcus mutans. Microbiology (Reading) 164:704–715. doi:10.1099/mic.0.000641 29504927 PMC5982141

[17] Nicod SS, Weinzierl ROJ, Burchell L, Escalera-Maurer A, James EH, Wigneshweraraj S. 2014. Systematic mutational analysis of the LytTR DNA binding domain of Staphylococcus aureus virulence gene transcription factor AgrA. Nucleic Acids Res 42:12523–12536. doi:10.1093/nar/gku1015 25352558 PMC4227749

[18] Mattos-Graner RO, Duncan MJ. 2017. Two-component signal transduction systems in oral bacteria. J Oral Microbiol 9:1400858. doi:10.1080/20002297.2017.1400858 29209465 PMC5706477

[19] Biswas I, Drake L, Erkina D, Biswas S. 2008. Involvement of sensor kinases in the stress tolerance response of Streptococcus mutans. J Bacteriol 190:68–77. doi:10.1128/JB.00990-07 17965153 PMC2223747

[20] Song L, Sudhakar P, Wang W, Conrads G, Brock A, Sun J, Wagner-Döbler I, Zeng A-P. 2012. A genome-wide study of two-component signal transduction systems in eight newly sequenced mutans streptococci strains. BMC Genomics 13:128. doi:10.1186/1471-2164-13-128 22475007 PMC3353171

[21] Kreth J, Merritt J, Zhu L, Shi W, Qi F. 2006. Cell density- and ComE-dependent expression of a group of mutacin and mutacin-like genes in Streptococcus mutans. FEMS Microbiol Lett 265:11–17. doi:10.1111/j.1574-6968.2006.00459.x 16981904

[22] Kreth J, Zhang Y, Herzberg MC. 2008. Streptococcal antagonism in oral biofilms: Streptococcus sanguinis and Streptococcus gordonii interference with Streptococcus mutans. J Bacteriol 190:4632–4640. doi:10.1128/JB.00276-08 18441055 PMC2446780

[23] Kreth J, Merritt J, Shi WY, Qi FX. 2005. Competition and coexistence between Streptococcus mutans and Streptococcus sanguinis in the dental biofilm. J Bacteriol 187:7193–7203. doi:10.1128/JB.187.21.7193-7203.2005 16237003 PMC1272965

[24] Srivastava SK, Rajasree K, Fasim A, Arakere G, Gopal B. 2014. Influence of the AgrC-AgrA complex on the response time of Staphylococcus aureus quorum sensing. J Bacteriol 196:2876–2888. doi:10.1128/JB.01530-14 24858185 PMC4135676

[25] Sun F, Liang H, Kong X, Xie S, Cho H, Deng X, Ji Q, Zhang H, Alvarez S, Hicks LM, Bae T, Luo C, Jiang H, He C. 2012. Quorum-sensing agr mediates bacterial oxidation response via an intramolecular disulfide redox switch in the response regulator AgrA. Proc Natl Acad Sci U S A 109:9095–9100. doi:10.1073/pnas.1200603109 22586129 PMC3384213

[26] Sidote DJ, Barbieri CM, Wu T, Stock AM. 2008. Structure of the Staphylococcus aureus AgrA LytTR domain bound to DNA reveals a beta fold with an unusual mode of binding. Structure 16:727–735. doi:10.1016/j.str.2008.02.011 18462677 PMC2430735

[27] Pinchas MD, LaCross NC, Dawid S. 2015. An electrostatic interaction between BlpC and BlpH dictates pheromone specificity in the control of bacteriocin production and immunity in Streptococcus pneumoniae. J Bacteriol 197:1236–1248. doi:10.1128/JB.02432-14 25622617 PMC4352655

[28] Valente C, Dawid S, Pinto FR, Hinds J, Simões AS, Gould KA, Mendes LA, de Lencastre H, Sá-Leão R. 2016. The blp locus of Streptococcus pneumoniae plays a limited role in the selection of strains that can cocolonize the human nasopharynx. Appl Environ Microbiol 82:5206–5215. doi:10.1128/AEM.01048-16 27316956 PMC4988185

[29] Wholey WY, Kochan TJ, Storck DN, Dawid S. 2016. Coordinated bacteriocin expression and competence in Streptococcus pneumoniae contributes to genetic adaptation through neighbor predation. PLoS Pathog 12:e1005413. doi:10.1371/journal.ppat.1005413 26840124 PMC4739721

[30] Ratner S, Bollinger K, Richardson J, Dawid S. 2022. The outer surface protease, SepM, is required for blp locus activation in three of the four most common pherotypes of Streptococcus pneumoniae. J Bacteriol 204:e0019622. doi:10.1128/jb.00196-22 36286514 PMC9664958

[31] Kreth J, Hung DCI, Merritt J, Perry J, Zhu L, Goodman SD, Cvitkovitch DG, Shi W, Qi F. 2007. The response regulator ComE in Streptococcus mutans functions both as a transcription activator of mutacin production and repressor of CSP biosynthesis. Microbiology (Reading) 153:1799–1807. doi:10.1099/mic.0.2007/005975-0 17526837 PMC2062498

[32] Hung DCI, Downey JS, Ayala EA, Kreth J, Mair R, Senadheera DB, Qi F, Cvitkovitch DG, Shi W, Goodman SD. 2011. Characterization of DNA binding sites of the ComE response regulator from Streptococcus mutans. J Bacteriol 193:3642–3652. doi:10.1128/JB.00155-11 21602345 PMC3133340

[33] Dhaked HPS, Cao L, Biswas I. 2021. Redox sensing modulates the activity of the ComE response regulator of Streptococcus mutans. J Bacteriol 203:e0033021. doi:10.1128/JB.00330-21 34516285 PMC8570269

[34] Ajdić D, McShan WM, McLaughlin RE, Savić G, Chang J, Carson MB, Primeaux C, Tian R, Kenton S, Jia H, Lin S, Qian Y, Li S, Zhu H, Najar F, Lai H, White J, Roe BA, Ferretti JJ. 2002. Genome sequence of Streptococcus mutans UA159, a cariogenic dental pathogen. Proc Natl Acad Sci U S A 99:14434–14439. doi:10.1073/pnas.172501299 12397186 PMC137901

[35] Biswas I, Jha JK, Fromm N. 2008. Shuttle expression plasmids for genetic studies in Streptococcus mutans. Microbiology (Reading, Engl) 154:2275–2282. doi:10.1099/mic.0.2008/019265-0 PMC4110107 18667560

[36] Bradford MM. 1976. A rapid and sensitive method for the quantitation of microgram quantities of protein utilizing the principle of protein-dye binding. Anal Biochem 72:248–254. doi:10.1016/0003-2697(76)90527-3 942051

[37] Bonfils C, Bec N, Lacroix B, Harricane MC, Larroque C. 2007. Kinetic analysis of tubulin assembly in the presence of the microtubule-associated protein TOGp. J Biol Chem 282:5570–5581. doi:10.1074/jbc.M605641200 17178729 PMC2238798

[38] Johnson KA, Borisy GG. 1977. Kinetic analysis of microtubule self-assembly in vitro. J Mol Biol 117:1–31. doi:10.1016/0022-2836(77)90020-1 599563

[39] Lange G, Mandelkow EM, Jagla A, Mandelkow E. 1988. Tubulin oligomers and microtubule oscillations. Antagonistic role of microtubule stabilizers and destabilizers. Eur J Biochem 178:61–69. doi:10.1111/j.1432-1033.1988.tb14429.x 3203694

[40] Stryer L. 1965. The interaction of a naphthalene dye with apomyoglobin and apohemoglobin. A fluorescent probe of non-polar binding sites. J Mol Biol 13:482–495. doi:10.1016/s0022-2836(65)80111-5 5867031

[41] Schonbrunn E, Eschenburg S, Luger K, Kabsch W, Amrhein N. 2000. Structural basis for the interaction of the fluorescence probe 8-anilino-1-naphthalene sulfonate (ANS) with the antibiotic target MurA. Proc Natl Acad Sci U S A 97:6345–6349. doi:10.1073/pnas.120120397 10823915 PMC18605

[42] Dhaked HPS, Ray S, Battaje RR, Banerjee A, Panda D. 2019. Regulation of Streptococcus pneumoniae FtsZ assembly by divalent cations: paradoxical effects of Ca^2+^ on the nucleation and bundling of FtsZ polymers. FEBS J 286:3629–3646. doi:10.1111/febs.14928 31090151

[43] Lan CY, Igo MM. 1998. Differential expression of the OmpF and OmpC porin proteins in Escherichia coli K-12 depends upon the level of active OmpR. J Bacteriol 180:171–174. doi:10.1128/JB.180.1.171-174.1998 9422609 PMC106865

[44] Stewart RC, Roth AF, Dahlquist FW. 1990. Mutations that affect control of the methylesterase activity of CheB, a component of the chemotaxis adaptation system in Escherichia coli. J Bacteriol 172:3388–3399. doi:10.1128/jb.172.6.3388-3399.1990 2188960 PMC209150

[45] Hung DCI, Downey JS, Kreth J, Qi F, Shi W, Cvitkovitch DG, Goodman SD. 2012. Oligomerization of the response regulator ComE from Streptococcus mutans is affected by phosphorylation. J Bacteriol 194:1127–1135. doi:10.1128/JB.06565-11 22210762 PMC3294772

[46] Klose KE, Weiss DS, Kustu S. 1993. Glutamate at the site of phosphorylation of nitrogen-regulatory protein NTRC mimics aspartyl-phosphate and activates the protein. J Mol Biol 232:67–78. doi:10.1006/jmbi.1993.1370 8331671

[47] Halfmann A, Schnorpfeil A, Müller M, Marx P, Günzler U, Hakenbeck R, Brückner R. 2011. Activity of the two-component regulatory system CiaRH in Streptococcus pneumoniae R6. J Mol Microbiol Biotechnol 20:96–104. doi:10.1159/000324893 21422763

[48] Wolfe AJ. 2005. The acetate switch. Microbiol Mol Biol Rev 69:12–50. doi:10.1128/MMBR.69.1.12-50.2005 15755952 PMC1082793

[49] Barbieri CM, Stock AM. 2008. Universally applicable methods for monitoring response regulator aspartate phosphorylation both in vitro and in vivo using Phos-tag-based reagents. Anal Biochem 376:73–82. doi:10.1016/j.ab.2008.02.004 18328252 PMC2504525

[50] Rivas G, López A, Mingorance J, Ferrándiz MJ, Zorrilla S, Minton AP, Vicente M, Andreu JM. 2000. Magnesium-induced linear self-association of the FtsZ bacterial cell division protein monomer. The primary steps for FtsZ assembly. J Biol Chem 275:11740–11749. doi:10.1074/jbc.275.16.11740 10766796

[51] Wyman C, Rombel I, North AK, Bustamante C, Kustu S. 1997. Unusual oligomerization required for activity of NtrC, a bacterial enhancer-binding protein. Science 275:1658–1661. doi:10.1126/science.275.5306.1658 9054362

[52] Gao R, Mack TR, Stock AM. 2007. Bacterial response regulators: versatile regulatory strategies from common domains. Trends Biochem Sci 32:225–234. doi:10.1016/j.tibs.2007.03.002 17433693 PMC3655528

[53] Sinha N, Nussinov R. 2001. Point mutations and sequence variability in proteins: redistributions of preexisting populations. Proc Natl Acad Sci U S A 98:3139–3144. doi:10.1073/pnas.051399098 11248045 PMC30620

[54] Kumar A, Biswas P. 2019. Effect of site-directed point mutations on protein misfolding: a simulation study. Proteins 87:760–773. doi:10.1002/prot.25702 31017329

[55] Dhaked HPS, Bhattacharya A, Yadav S, Dantu SC, Kumar A, Panda D. 2016. Mutation of Arg191 in FtsZ impairs cytokinetic abscission of Bacillus subtilis cells. Biochemistry 55:5754–5763. doi:10.1021/acs.biochem.6b00493 27629358

[56] Liu W, Hulett FM. 1997. Bacillus subtilis PhoP binds to the phoB tandem promoter exclusively within the phosphate starvation-inducible promoter. J Bacteriol 179:6302–6310. doi:10.1128/jb.179.20.6302-6310.1997 9335276 PMC179543

[57] Wösten MM, Groisman EA. 1999. Molecular characterization of the PmrA regulon. J Biol Chem 274:27185–27190. doi:10.1074/jbc.274.38.27185 10480935

[58] Toro-Roman A, Mack TR, Stock AM. 2005. Structural analysis and solution studies of the activated regulatory domain of the response regulator ArcA: a symmetric dimer mediated by the α4-β5-α5 face. J Mol Biol 349:11–26. doi:10.1016/j.jmb.2005.03.059 15876365 PMC3690759

[59] Ibrahim IM, Rowden SJL, Cramer WA, Howe CJ, Puthiyaveetil S. 2022. Thiol redox switches regulate the oligomeric state of cyanobacterial Rre1, RpaA and RpaB response regulators. FEBS Lett 596:1533–1543. doi:10.1002/1873-3468.14340 35353903 PMC9321951

[60] Fiedler U, Weiss V. 1995. A common switch in activation of the response regulators NtrC and PhoB: phosphorylation induces dimerization of the receiver modules. EMBO J 14:3696–3705. doi:10.1002/j.1460-2075.1995.tb00039.x 7641688 PMC394444

[61] Cvitkovitch DG. 2001. Genetic competence and transformation in oral streptococci. Crit Rev Oral Biol Med 12:217–243. doi:10.1177/10454411010120030201 11497374

[62] Li YH, Hanna MN, Svensäter G, Ellen RP, Cvitkovitch DG. 2001. Cell density modulates acid adaptation in Streptococcus mutans: implications for survival in biofilms. J Bacteriol 183:6875–6884. doi:10.1128/JB.183.23.6875-6884.2001 11698377 PMC95529

[63] Li Y-H, Tang N, Aspiras MB, Lau PCY, Lee JH, Ellen RP, Cvitkovitch DG. 2002. A quorum-sensing signaling system essential for genetic competence in Streptococcus mutans is involved in biofilm formation. J Bacteriol 184:2699–2708. doi:10.1128/JB.184.10.2699-2708.2002 11976299 PMC135014

[64] Li Y-H, Lau PCY, Lee JH, Ellen RP, Cvitkovitch DG. 2001. Natural genetic transformation of Streptococcus mutans growing in biofilms. J Bacteriol 183:897–908. doi:10.1128/JB.183.3.897-908.2001 11208787 PMC94956

[65] Dhaked HPS, Biswas I. 2022. Distribution of two-component signal transduction systems BlpRH and ComDE across streptococcal species. Front Microbiol 13:960994. doi:10.3389/fmicb.2022.960994 36353461 PMC9638458

[66] Shankar M, Hossain MS, Biswas I. 2017. Pleiotropic regulation of virulence genes in Streptococcus mutans by the conserved small protein SprV. J Bacteriol 199:e00847-16. doi:10.1128/JB.00847-16 28167518 PMC5370425

[67] Levin JC, Wessels MR. 1998. Identification of csrR/csrS, a genetic locus that regulates hyaluronic acid capsule synthesis in group A Streptococcus. Mol Microbiol 30:209–219. doi:10.1046/j.1365-2958.1998.01057.x 9786197

[68] Froehlich BJ, Bates C, Scott JR. 2009. Streptococcus pyogenes CovRS mediates growth in iron starvation and in the presence of the human cationic antimicrobial peptide LL-37. J Bacteriol 191:673–677. doi:10.1128/JB.01256-08 18996992 PMC2620807

[69] Federle MJ, Scott JR. 2002. Identification of binding sites for the group A streptococcal global regulator CovR. Mol Microbiol 43:1161–1172. doi:10.1046/j.1365-2958.2002.02810.x 11918804

[70] Biswas S, Biswas I. 2006. Regulation of the glucosyltransferase (gtfBC) operon by CovR in Streptococcus mutans. J Bacteriol 188:988–998. doi:10.1128/JB.188.3.988-998.2006 16428403 PMC1347363

[71] Chong P, Drake L, Biswas I. 2008. Modulation of covR expression in Streptococcus mutans UA159. J Bacteriol 190:4478–4488. doi:10.1128/jb.01961-07 18469111 PMC2446802

[72] Ulijasz AT, Andes DR, Glasner JD, Weisblum B. 2004. Regulation of iron transport in Streptococcus pneumoniae by RitR, an orphan response regulator. J Bacteriol 186:8123–8136. doi:10.1128/JB.186.23.8123-8136.2004 15547286 PMC529065

[73] Antoraz S, Rico S, Rodríguez H, Sevillano L, Alzate JF, Santamaría RI, Díaz M. 2017. The orphan response regulator Aor1 is a new relevant piece in the complex puzzle of Streptomyces coelicolor antibiotic regulatory network. Front Microbiol 8:2444. doi:10.3389/fmicb.2017.02444 29312165 PMC5733086

[74] Gueriri I, Cyncynatus C, Dubrac S, Arana AT, Dussurget O, Msadek T. 2008. The DegU orphan response regulator of Listeria monocytogenes autorepresses its own synthesis and is required for bacterial motility, virulence and biofilm formation. Microbiology (Reading) 154:2251–2264. doi:10.1099/mic.0.2008/017590-0 18667558

[75] Overgaard M, Wegener-Feldbrügge S, Søgaard-Andersen L. 2006. The orphan response regulator DigR is required for synthesis of extracellular matrix fibrils in Myxococcus xanthus. J Bacteriol 188:4384–4394. doi:10.1128/JB.00189-06 16740945 PMC1482965

[76] Black WP, Wang L, Davis MY, Yang Z. 2015. The orphan response regulator EpsW is a substrate of the DifE kinase and it regulates exopolysaccharide in Myxococcus xanthus. Sci Rep 5:17831. doi:10.1038/srep17831 26639551 PMC4671073

[77] Bauer S, Endres M, Lange M, Schmidt T, Schumbrutzki C, Sickmann A, Beier D. 2013. Novel function assignment to a member of the essential HP1043 response regulator family of epsilon-proteobacteria. Microbiology (Reading) 159:880–889. doi:10.1099/mic.0.066548-0 23475951

[78] Rowland MA, Deeds EJ. 2014. Crosstalk and the evolution of specificity in two-component signaling. Proc Natl Acad Sci U S A 111:5550–5555. doi:10.1073/pnas.1317178111 24706803 PMC3992699

[79] Laub MT, Goulian M. 2007. Specificity in two-component signal transduction pathways. Annu Rev Genet 41:121–145. doi:10.1146/annurev.genet.41.042007.170548 18076326

[80] Wanner BL. 1992. Is cross regulation by phosphorylation of two-component response regulator proteins important in bacteria? J Bacteriol 174:2053–2058. doi:10.1128/jb.174.7.2053-2058.1992 1551826 PMC205819

[81] Vemparala B, Valiya Parambathu A, Saini DK, Dixit NM. 2022. An evolutionary paradigm favoring cross talk between bacterial two-component signaling systems. mSystems 7:e0029822. doi:10.1128/msystems.00298-22 36264076 PMC9765234

[82] Reck M, Tomasch J, Wagner-Döbler I. 2015. The alternative sigma factor SigX controls bacteriocin synthesis and competence, the two quorum sensing regulated traits in Streptococcus mutans. PLoS Genet 11:e1005353. doi:10.1371/journal.pgen.1005353 26158727 PMC4497675

[83] Perry JA, Cvitkovitch DG, Lévesque CM. 2009. Cell death in Streptococcus mutans biofilms: a link between CSP and extracellular DNA. FEMS Microbiol Lett 299:261–266. doi:10.1111/j.1574-6968.2009.01758.x 19735463 PMC2771664

[84] Grebe TW, Stock JB. 1999. The histidine protein kinase superfamily. Adv Microb Physiol 41:139–227. doi:10.1016/s0065-2911(08)60167-8 10500846

[85] Dutta R, Inouye M. 2000. GHKL, an emergent ATPase/kinase superfamily. Trends Biochem Sci 25:24–28. doi:10.1016/s0968-0004(99)01503-0 10637609

[86] Agrawal R, Sahoo BK, Saini DK. 2016. Cross-talk and specificity in two-component signal transduction pathways. Future Microbiol 11:685–697. doi:10.2217/fmb-2016-0001 27159035

[87] Schär J, Sickmann A, Beier D. 2005. Phosphorylation-independent activity of atypical response regulators of Helicobacter pylori. J Bacteriol 187:3100–3109. doi:10.1128/JB.187.9.3100-3109.2005 15838037 PMC1082831

[88] Servetas SL, Carpenter BM, Haley KP, Gilbreath JJ, Gaddy JA, Merrell DS. 2016. Characterization of key Helicobacter pylori regulators identifies a role for ArsRS in biofilm formation. J Bacteriol 198:2536–2548. doi:10.1128/JB.00324-16 27432830 PMC4999924

[89] Galperin MY. 2008. Telling bacteria: do not LytTR. Structure 16:657–659. doi:10.1016/j.str.2008.04.003 18462668 PMC2724992

[90] Zou Z, Qin H, Brenner AE, Raghavan R, Millar JA, Gu Q, Xie Z, Kreth J, Merritt J. 2018. LytTR regulatory systems: a potential new class of prokaryotic sensory system. PLoS Genet 14:e1007709. doi:10.1371/journal.pgen.1007709 30296267 PMC6193735

