# Supplementary material for: A novel insight into ComE-mediated activation of gene expression in Streptococcus mutans
Source: Microbiol Spectr. 2025 Jul 7;13(8):e01477-25. doi: 10.1128/spectrum.01477-25 (PMC12323615; doi:10.1128/spectrum.01477-25)
Supplement: Supplemental figures — Figures S1 and S2. [file spectrum.01477-25-s0001.pdf]

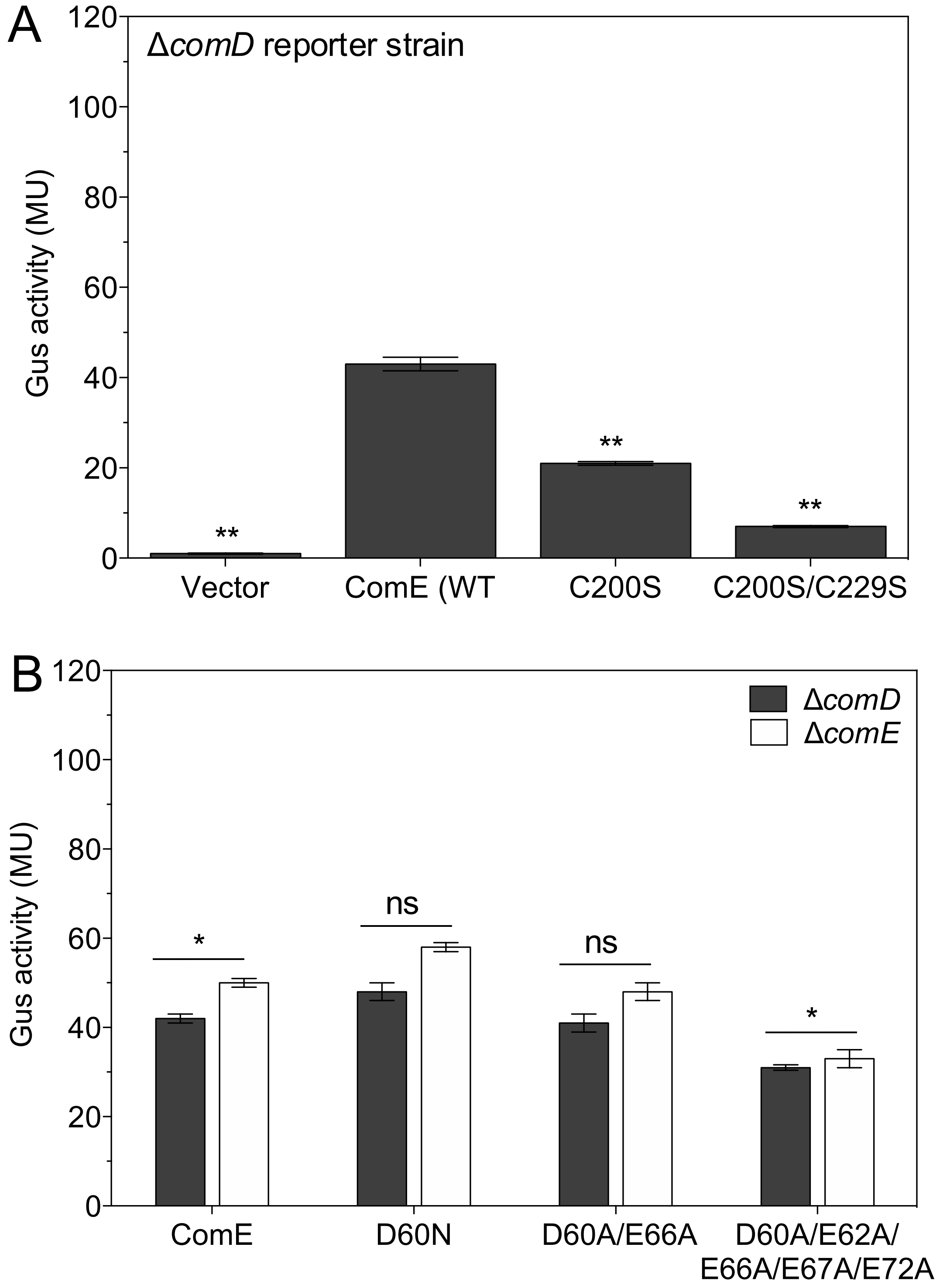

Figure S1. (A) The β-Glucuronidase (Gus) assays of Δ*comD* reporter strains containing empty vector pIB184Em or vector containing ComE (WT), C200S, and C200S/C229S were performed. (B) The Gus assays of Δ*comD* and Δ*comE* reporter strains containing vector with ComE (WT), D60N, D60A/E66A, and D60A/E62A/E66A/E67A/E72A were performed.

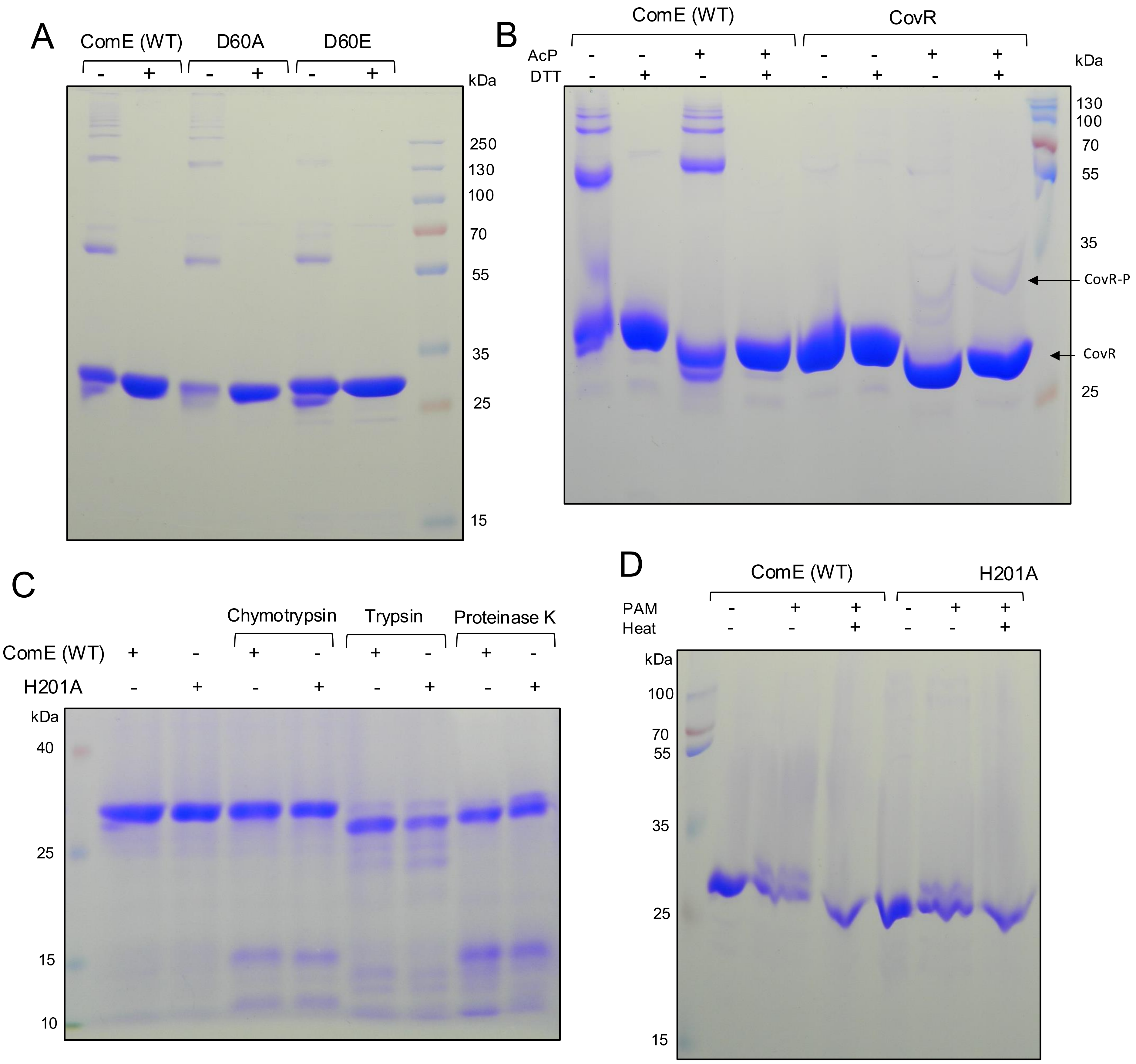

Figure S2. (A) Isolated ComE (WT) and phosphovariants (D60A and D60E) were incubated without (-) or with (+) 5 mM DTT for 30 min at room temperature. Samples were loaded on a 12% non-reducing (no DTT) SDS-PAGE. (B) Isolated proteins were incubated without and with 5 mM DTT at room temperature for 30 min in 1X buffer (20 mM Tris, pH7.4, 20 mM MgCl2, 50 mM KCl, 10 % glycerol). Then each reaction was incubated in the absence and presence of 50 mM acetyl phosphate for additional 1 hr. The samples were loaded on 12 % Phos-tag SDS PAGE. (C) Isolated proteins were mixed in 1X buffer (20 mM Tris, pH7.4, 200 mM KCl, 10 % glycerol) containing 5 mM DTT. These reduced proteins were incubated with 0.5 µM chymotrypsin, 0.1 µM trypsin, and 0.1 µM proteinase K at 4 °C for 10 min. The reaction was quenched using 10 mM PMSF. The samples were loaded on 12 % SDS PAGE. Experiment was performed two times. (D) The isolated reduced proteins were processed for phosphorylation as mentioned in text and loaded on 12 % Phos-tag SDS PAGE. Experiment was performed at least twice.
